# Respiratory viral infections and effects of meteorological parameters and air pollution in adults with respiratory symptoms admitted to the emergency room

**DOI:** 10.1111/irv.12158

**Published:** 2013-08-26

**Authors:** Denise R Silva, Vinícius P Viana, Alice M Müller, Fernando P Livi, Paulo de Tarso R Dalcin

**Affiliations:** a Pulmonology Department, School of Medicine, Graduate Program in Pulmonology, Universidade Federal do Rio Grande do SulPorto Alegre, RS, Brazil; b Geoscience Institute, School of Geography, Universidade Federal do Rio Grande do SulPorto Alegre, RS, Brazil

**Keywords:** Air pollution, hospitalizations, influenza-like illness, meteorology, respiratory viral infections, severe acute respiratory infections

## Abstract

**Background:**

Respiratory viral infections (RVIs) are the most common causes of respiratory infections. The prevalence of respiratory viruses in adults is underestimated. Meteorological variations and air pollution are likely to play a role in these infections.

**Objectives:**

The objectives of this study were to determine the number of emergency visits for influenza-like illness (ILI) and severe acute respiratory infection (SARI) and to evaluate the association between ILI/SARI, RVI prevalence, and meteorological factors/air pollution, in the city of Porto Alegre, Brazil, from November 2008 to October 2010.

**Methods:**

Eleven thousand nine hundred and fifty-three hospitalizations (adults and children) for respiratory symptoms were correlated with meteorological parameters and air pollutants. In a subset of adults, nasopharyngeal aspirates were collected and analyzed through IFI test. The data were analyzed using time-series analysis.

**Results:**

Influenza-like illness and SARI were diagnosed in 3698 (30·9%) and 2063 (17·7%) patients, respectively. Thirty-seven (9·0%) samples were positive by IFI and 93 of 410 (22·7%) were IFI and/or PCR positive. In a multivariate logistic regression model, IFI positivity was statistically associated with absolute humidity, use of air conditioning, and presence of mold in home. Sunshine duration was significantly associated with the frequency of ILI cases. For SARI cases, the variables mean temperature, sunshine duration, relative humidity, and mean concentration of pollutants were singnificant.

**Conclusions:**

At least 22% of infections in adult patients admitted to ER with respiratory complaints were caused by RVI. The correlations among meteorological variables, air pollution, ILI/SARI cases, and respiratory viruses demonstrated the relevance of climate factors as significant underlying contributors to the prevalence of RVI.

## Introduction

Respiratory tract infections are the most common causes of infection, and viruses account for the majority of these infections, leading to significant levels of morbidity and mortality.^1^ Emergency rooms (ERs) serve as the frontline for patients at highest risk for respiratory infection diseases, especially because of the acute nature of these illnesses.^2^

The prevalence of respiratory viral infection (RVI) in adults admitted to the ER is largely unexplored, as most relevant data concern infants and children.^3^,^4^ RVI can be severe in elderly patients, especially in those with underlying respiratory or cardiac disease. During winter months, RVI can account for many of the admissions to hospitals.^5^,^6^ The etiology of respiratory infections in adults remains undetermined in more than 50% of cases.^7^,^8^ In a study with 510 adults hospitalized with pulmonary diseases, an overall prevalence of respiratory viruses (RVs) in the lower respiratory tract was of 42·2%, with rhinoviruses and influenza A virus being the most common.^9^ In adults with acute asthma admitted to the ER, a prevalence of 12·2% of RVI was found.^10^ In another study, with adults admitted to hospital with respiratory symptoms, viruses accounted for 15% of hospital admissions for respiratory infections.^11^

Seasonality of certain acute respiratory tract infection pathogens can be explained by meteorological variations. In a study, temperature was highly inversely correlated with respiratory syncytial virus (RSV), influenza A, and adenovirus frequency; rhinovirus was also associated with relative humidity (RH). Climatic factors may influence the interaction among the host, pathogen, and environment, increasing the probability of exposure, susceptibility, and infection.^12^ In addition, experimental data have shown that air pollutants affect lung immune responses and inflammatory reactions and that these effects may underlie the increased risk for respiratory infections.^13^,^14^

Because of the large impact respiratory virus infections have on morbidity and even mortality, it is important to understand whether and how meteorological factors and exposure to air pollutants could influence respiratory virus infections. The aims of this study were to determine the number of emergency room visits for influenza-like illness (ILI) and severe acute respiratory infection (SARI) and to evaluate the association between ILI/SARI frequency, respiratory virus prevalence, and meteorological factors/air pollution, especially in adult population, in a humid subtropical climate.

## Methods

The present study was divided into two parts: in the first one, we characterized the symptomatic respiratory subjects attending the ER at Hospital de Clínicas de Porto Alegre (HCPA), during 1 year (November 2008 to October 2009). In the second part, we included patients with respiratory symptoms ≤5 days to determine the prevalence of RVI; this part was conducted during 2 years (November 2008 to October 2010). In the first year of the study, we also collected climate and air pollution data.

### Study setting

The study was conducted at HCPA, in the city of Porto Alegre, southern Brazil. The HCPA is a general, tertiary care, university-affiliated hospital with 750 beds and approximately 30 000 hospitalizations/year. With a population of 1 360 590 inhabitants, Porto Alegre is surrounded by a metropolitan area that encompasses 31 municipalities (3 717 430 inhabitants). Porto Alegre has a humid subtropical climate, with the hallmark of the great variability (classification Cfa in Köppen–Geiger).^15^ The ethics committee at HCPA has approved access to patient records. All subjects selected for the study gave written informed consent to participate.

### Clinical data

In the first part of the study, the clinical records of all daily visits (adults ≥18 years and children) to the ER were reviewed by the research team. Patients with respiratory symptoms (cough, coryza, nasal obstruction, odynophagia, dyspnea, chest pain, dysphonia, wheezing, and fever), regardless of the onset time, were included in the study. Patients presenting only with fever were not included. Demographic, clinical, and laboratorial characteristics were registered in a standardized questionnaire: sex, age, race, years of schooling, smoking status, symptoms at admission, duration of symptoms, presence of comorbidities, admission vital signs (temperature, heart rate, respiratory rate, and peripheral oxygen saturation measured by a digital oximeter), breath sounds, radiological findings, length of hospital stay, intensive care unit (ICU) admission, hospitalization outcome (death or discharge).

In the second part, adults ≥18 years with respiratory symptoms ≤5 days were included, and nasopharyngeal aspirates were collected. Every day, a daily shift (morning, afternoon, or evening) was randomized for inclusion of patients. The patients were interviewed by a member of the research team, and the following data were registered in a standardized questionnaire (in addition to the data already registered in the first part): family income, influenza vaccine, previous antibiotic use, and occupational exposure, flu symptoms in the family, presence of smokers at home, family history of respiratory disease, air conditioning at home or at work, use of wood stoves, mold in home.

The total cases of ILI (fever >38°C and cough or sore throat) and SARI (fever >38°C and cough or sore throat and shortness of breath or difficulty breathing) were registered.

### Laboratory data

Nasopharyngeal aspirates were obtained according to a standard protocol for the second part of study. An indirect immunofluorescence assay (IFI) was carried out using the Respiratory Panel 1 Viral Screening & Identification Kit (Chemicon International, Temecula, CA, USA) for influenza A, influenza B, RSV, parainfluenza virus (PIV) types 1–3, and adenovirus. An aliquot of the aspirate was stored in liquid nitrogen for subsequent RT-PCR. The kit Seeplex® RV 12 ACE Detection (Seegene, Seoul, Korea) was used to detect 12 respiratory viruses (adenovirus, human metapneumovirus [hMPV], coronavirus [hCoV] 229E/NL63, hCoV OC43/HKU1, PIV 1, 2, and 3, influenza A and B, RSV A and B and rhinovirus A/B), according to the manufacturer's instructions.

### Meteorological data

Daily meteorological parameters, like temperature (maximum, average, and minimum, in °C), relative (%) and absolute (g/kg) humidity, rainfall (mm), and sunshine duration (number of sunshine hours per day), were obtained from the Climate Laboratory at the Federal University of Rio Grande do Sul. Also, the mean concentration of pollutants (μm/m^3^) was recorded. The methodology used by this laboratory informs automatically the pollutant that reached the highest concentration in the last 24 hours. In the city of Porto Alegre, it is probable that the pollutant that is responsible for more than 90% of the days is ozone, and in the rest of days, particulate matter of <10 μm dynamic diameter (PM_10_).

### Statistical analysis

Data were presented as number of cases, mean ± standard deviation (SD), or median with interquartile range (IQR). Categorical comparisons were carried out by chi-square test using Yates's correction if indicated or by Fisher's exact test. Continuous variables were compared using the *t*-test or Wilcoxon test. Odds ratios (ORs) and nominal 95% confidence intervals (CI) were presented. A two-sided *P* value <0·05 was considered significant for all analyses. Data analysis was performed using spss 18·0 (Statistical Package for the Social Sciences, Chicago, IL, USA) and the free statistical software R (http://www.r-project.org).

The data were analyzed using time-series analysis, through a generalized linear model (GLM) to examine the association between ILI or SARI and air pollution/meteorological variables, using logistic regression. An autoregressive integrated moving average (ARIMA) model was developed for ILI or SARI. Pollution and meteorological parameters were inserted as explanatory variables into these ARIMA models. For the second part of the study, further models were explored to include IFI and/or PCR results as outcome variables. Volatility of the mean model variance error(s) was addressed using Autoregressive Conditional Heteroscedasticity (ARCH) models within an ARIMA modeling framework.

An ARIMA was developed for ILI and SARI. ARIMA (p, d, q) are useful tools for analyzing time-series data containing non-stationary common trends, and these models were proposed by Box and Jenkins in 1976. The ARIMA (p, d, q) allow to make predictions from their parties AR (autoregressive) and MA (moving average). For both SG and SARS, we made the identification of the order of the parts and autoregressive moving average following the Box–Jenkins approach. First we identified the need for differentiation, for ILI and SARI, the graphical analysis of time-series and autocorrelation functions, as well as for testing the unit root (Dickey–Fuller) who did not reject the hypothesis of non-stationarity for ILI and SARI. In a second moment, it was noted that there was no seasonality for ILI and SARI, by analyzing the periodograms and autocorrelation functions and partial autocorrelation. Subsequently, variable selection models ARIMA (p, d, q) for ILI and SARI followed the approach backward. The Akaike information criterion (AIC) – which acts to penalize the number of parameters in the model – and the Schwarz information criterion (SIC) (or Bayesian information criterion, BIC) were also used to select models.

Sample size requirements were estimated from the literature review.^10^,^11^ Using a prevalence of RVI in adults of 15%, with a significance level of 99%, and total confidence interval amplitude of 0·10, we calculated that 339 patients would be needed in the second part of the study.

## Results

During the 12-month study period, there were 37 059 admissions to the ER (24 189 adults and 12 870 children), of which 11 953 (32·3%) presented with respiratory symptoms. The most common symptoms were cough (73·4%), fever (56·1%), dyspnea (40·9%), chest pain (24·5%), and coryza (20·9%). The median duration of symptoms before admission was 3 days (IQR: 1–6 days). A total of 2205 (18·5%) patients admitted to ER needed to be hospitalized; of these patients, 242 (2·0%) required ICU admission. ILI and SARI were diagnosed in 3698 (30·9%) and 2063 (17·7%) patients, respectively. The overall mortality rate among all study participants was 280 of 11 953 (2·3%). Demographic and clinical characteristics of the study population are shown in Table 1.

**Table 1:** Characteristics of patients with respiratory symptoms (November 2008–2009; *n* = 11 953)*

Characteristics	Adults (*n* = 6546)	Children (*n* = 5407)
Age, year	50·6 ± 19·2	2 (0·01–17)
Male sex	2942 (45·0)	2977 (55·1)
White race	5593 (85·6)	4336 (80·3)
<8 year of schooling	2764 (47·8)	–
Current smokers	609 (9·3)	–
Cough	4265 (65·2)	4501 (83·3)
Fever	2708 (41·4)	3994 (73·9)
Dyspnea/tachypnea	3401 (52·0)	1482 (27·4)
Chest pain	2583 (39·5)	343 (6·3)
Coryza	615 (9·4)	1882 (34·8)
Sore throat	767 (11·7)	602 (11·1)
Duration of symptoms before admission	3 (1–6)	2 (1–4)
ILI	1321 (20·2)	2376 (44·0)
SARI	845 (12·9)	1218 (22·5)
Need for hospitalization	1526 (23·3)	679 (12·6)
Mortality rate	260 (4·0)	20 (0·4)

ILI, influenza-like illness; SARI, severe acute respiratory infection.

* Data are presented as mean ± SD, *n*/*N* (%): number of cases with characteristic/total number of cases (percentage) or median (interquartile range).

According to the selection criteria of the study, 425 patients met the inclusion criteria and were invited to participate, and 15 patients declined to participate. Then, 410 adults were enrolled for virological investigation, 255 in the first year and 155 in the second one. There were no differences in age, sex, race, years of schooling, and symptoms between selected sample and all adults admitted to the ER in the same period. However, as expected, the median duration of symptoms was lower in the selected sample as compared to all adults admitted to the ER [3·0 days (2·0–4·0) versus 3·0 days (1·0–7·0); *P* < 0·0001]. Thirty-seven (9·0%) samples were positive by IFI: 11 influenza A, 11 RSV, 8 PIV type 3, 3 adenovirus, 2 PIV type 2, 1 PIV type 1, and 1 influenza B. The characteristics of IFI-positive and IFI-negative patients were described in Table 2.

**Table 2:** Characteristics of patients with IFI positive and IFI negative (November 2008–2009; *n* = 410)*

Characteristics	IFI positive (*n* = 37)	IFI negative (*n* = 373)	*P* value
Age, year	50·4 ± 19·5	51·4 ± 18·4	0·741
Male sex	16 (43·2)	148 (39·7)	0·673
White race	29 (78·4)	269 (72·1)	0·415
Years of schooling	8 (5–11)	7 (4–10)	0·139
Current smokers	7 (31·8)	78 (37·1)	0·622
Presence of smokers at home	12 (32·4)	141 (37·8)	0·520
Air conditioning at home or at work	13 (35·1)	68 (18·2)	0·014
Use of wood stoves	6 (16·2)	51 (13·7)	0·670
Mold in home	15 (40·5)	118 (31·6)	0·270
Cough	36 (97·3)	315 (84·5)	0·034
Fever	24 (64·9)	190 (50·9)	0·106
Dyspnea	32 (86·5)	314 (84·2)	0·713
Chest pain	28 (75·7)	233 (62·5)	0·111
Coryza	20 (5·1)	171 (46·0)	0·347
Sore throat	8 (21·6)	94 (25·2)	0·631
Wheezing	27 (73·0)	199 (53·4)	0·022
Duration of symptoms before admission	2 (2–4)	3 (2–4)	0·613
ILI	10 (27·0)	43 (11·5)	0·007
SARI	23 (62·2)	143 (38·3)	0·005
Need for hospitalization	9 (24·3)	96 (25·8)	0·931
Length of hospital stay, days	4 (2–5)	6 (3–15·8)	0·033
Mortality rate	1 (2·7)	5 (1·3)	0·342

ILI, influenza-like illness; SARI, severe acute respiratory infection.

* Data are presented as mean ± SD, *n*/*N* (%): number of cases with characteristic/total number of cases (percentage) or median (interquartile range).

**Figure 1:**
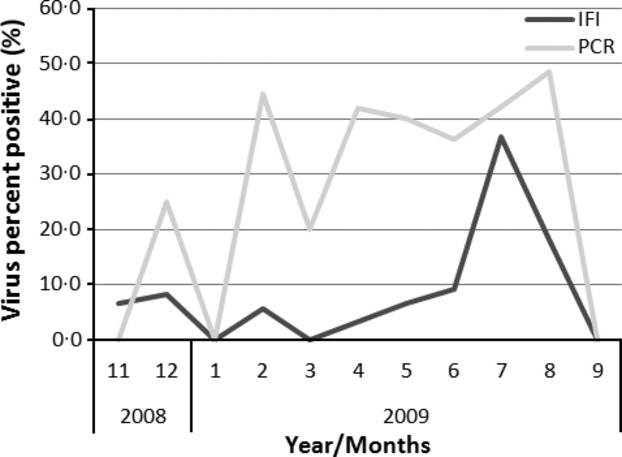
Times-series graph for virus percent positive by IFI and by PCR by month (IFI: November 2008–2009; *n* = 255 and PCR: November 2008 to September 2009; *n* = 180).

Samples from 180 patients were sent for RT-PCR analysis. Sixty-five (36·1%) samples were positive, with 70 viruses being identified (five patients had two viruses): 26 influenza A (with 15 H1N1), 19 rhinovirus, eight hCoV 229E/NL63, five PIV type 3, three hMPV, two adenovirus, two PIV type 1, two RSV B, one RSV A, one PIV type 2, and one PIV type 4. Seven samples considered to be unsatisfactory for IFI were positive by PCR. Another 50 samples that were negative in IFI were positive by PCR. Of the 37 samples positive in IFI, 18 were sent for PCR too, and in nine, the result was positive. In seven of these cases, the results of IFI and PCR were concordant. In one patient, adenovirus was identified on IFI and influenza A was found in PCR. In another case, IFI detected PIV type 2, and PCR identified PIV types 1 and 3. The characteristics of patients with PCR positive and negative were shown in Table 3. Figure 1 shows times-series graph for virus percent positive by IFI and by PCR by month.

**Table 3:** Characteristics of patients with PCR positive and negative (November 2008 to September 2009; *n* = 180)*

Characteristics	PCR positive (*n* = 65)	PCR negative (*n* = 115)	*P* value
Age, year	48·3 ± 20·1	51·7 ± 18·3	0·248
Male sex	24 (36·9)	44 (38·3)	0·986
White race	48 (73·8)	87 (75·7)	0·929
Years of schooling	8 (5·0–11·0)	8 (4·5–11·0)	0·929
Current smokers	22 (36·7)	20 (20·2)	0·036
Presence of smokers at home	29 (44·6)	39 (33·9)	0·207
Air conditioning at home or at work	17 (26·2)	15 (13·0)	0·045
Use of wood stoves	5 (7·7)	15 (13·0)	0·395
Mold in home	17 (26·2)	37 (32·2)	0·498
Cough	59 (90·8)	99 (86·1)	0·494
Fever	43 (66·2)	66 (57·4)	0·319
Dyspnea	53 (81·5)	101 (87·8)	0·351
Chest pain	43 (66·2)	71 (61·7)	0·668
Coryza	34 (52·3)	49 (43·0)	0·295
Sore throat	12 (18·5)	25 (21·7)	0·741
Wheezing	30 (46·2)	68 (59·1)	0·128
Duration of symptoms before admission	3 (1·25-4·0)	3 (2·0-4·0)	0·837
ILI	17 (26·2)	16 (13·9)	0·066
SARI	32 (49·2)	52 (45·2)	0·717
Need for hospitalization	7 (10·8)	32 (27·8)	0·013
Length of hospital stay, days	0·5 (0·5–2·5)	2 (0·5–6·0)	0·008
Mortality rate	0 (0)	2 (1·7)	0·536

ILI, influenza-like illness; SARI, severe acute respiratory infection.

* Data are presented as mean ± SD, *n*/*N* (%): number of cases with characteristic/total number of cases (percentage), or median (interquartile range).

**Figure 2:**
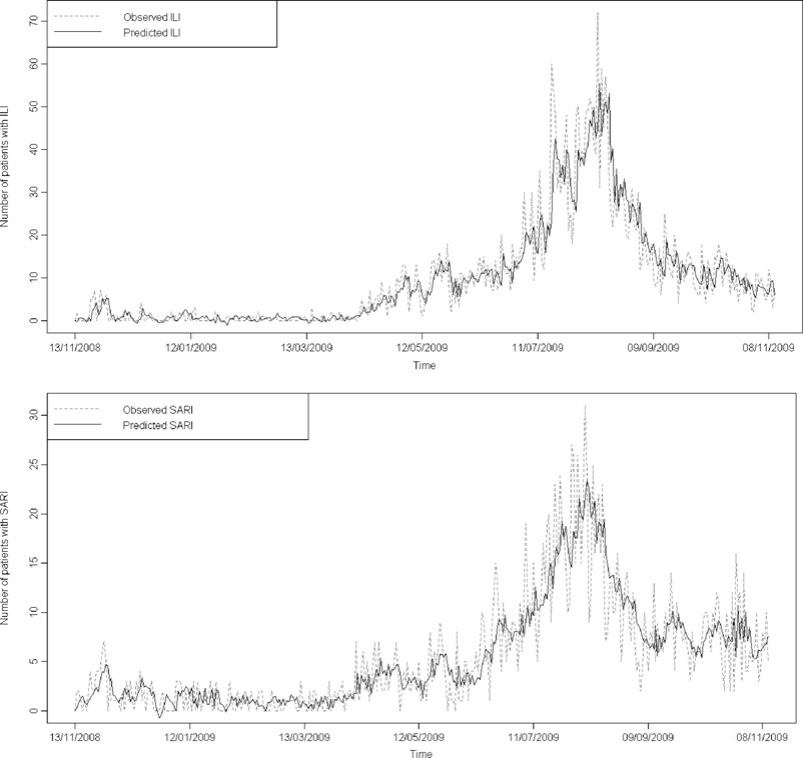
Modeled and observed values for ILI and SARI cases (ILI: November 2008–2009; *n* = 1·321 and SARI: November 2008–2009; *n* = 845).

**Figure 3:**
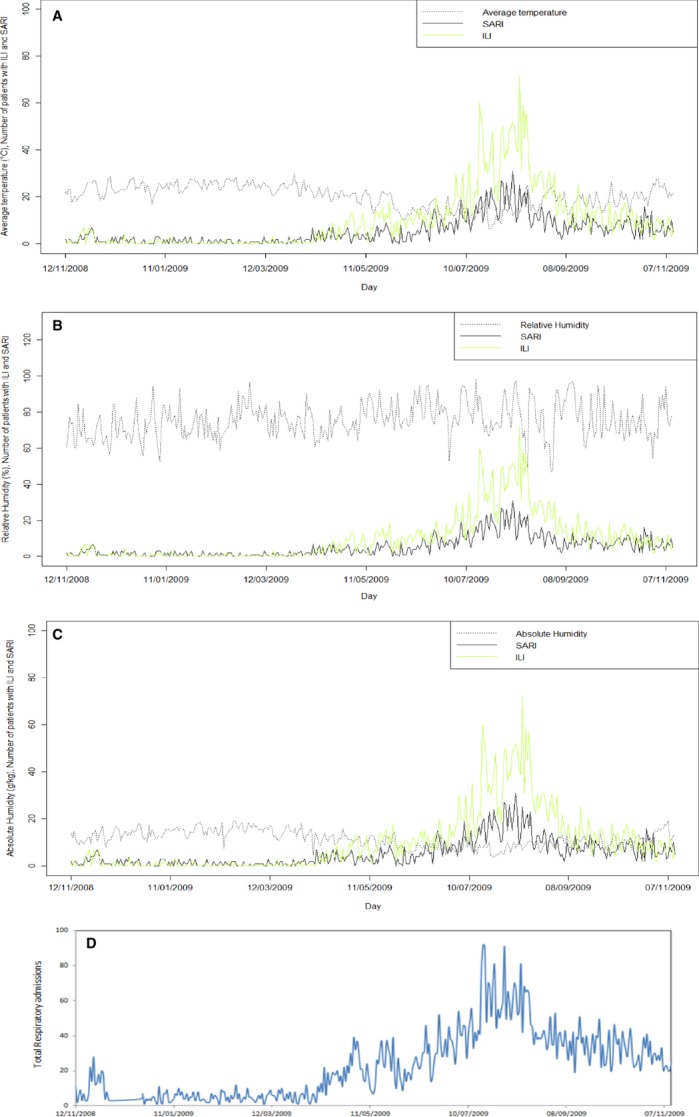
Daily number of patients with ILI and SARI against (A) daily average temperature, (B) daily absolute humidity, and (C) relative humidity. D shows daily total respiratory admissions (ILI: November 2008–2009; *n* = 1·321 and SARI: November 2008–2009; *n* = 845).

**Figure 4:**
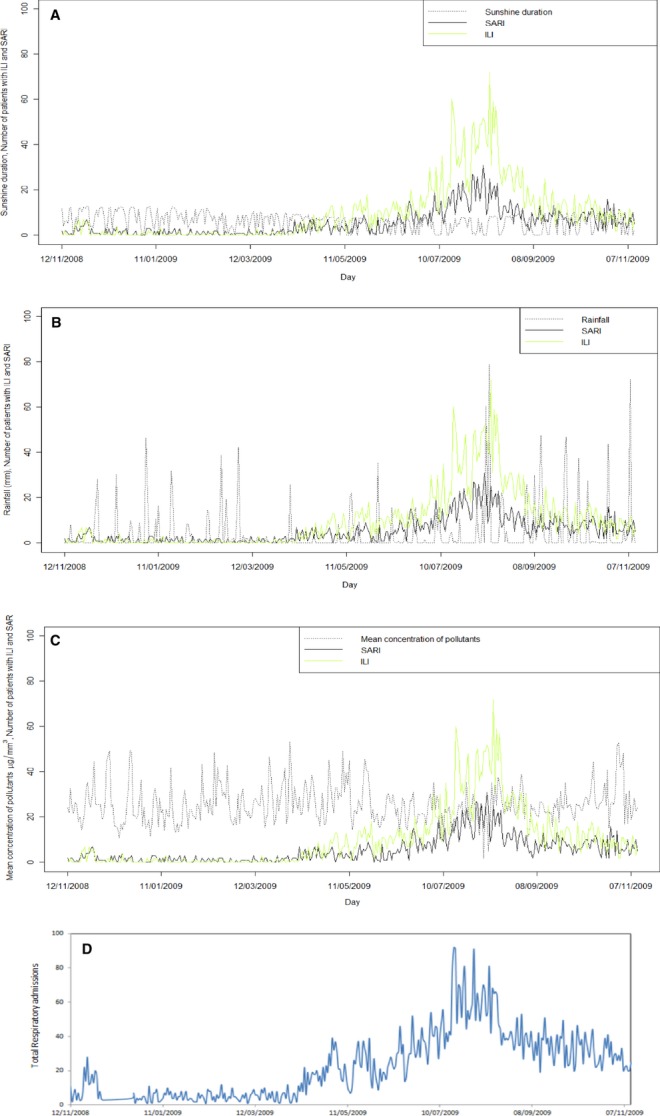
Daily number of patients with ILI and SARI against (A) sunshine duration, (B) rainfall, and (C) mean concentration of pollutants. (D) shows daily total respiratory admissions (ILI: November 2008–2009; *n* = 1·321 and SARI: November 2008–2009; *n* = 845).

Table 4 shows the descriptive statistics corresponding to the environmental variables considered in this study. Figure 2 shows the modeled and observed values for ILI and SARI cases. Figures3 and 4 show the daily number of patients with ILI and SARI and meteorological parameters. We checked the autocorrelation between the covariates of climate data and observations from previous days, and we considered the following lags (in days) for each variable: average temperature (5), rainfall (2), sunshine duration (6), relative humidity (5), mean concentration of pollutants (3), and absolute humidity (2).

**Table 4:** Statistics for environmental variables (November 2008–2009)

Variables	Minimum	Maximum	Mean	Standard deviation
Tmax (°C)	10·5	39·6	25·5	5·6
Tmin (°C)	0	24·0	15·6	4·7
Absolute humidity (g/kg)	3·8	19·3	11·8	3·5
Relative humidity (%)	47·0	98·3	75·8	9·6
Rainfall (mm)*	0	79·0	4·3	10·9
Sunshine duration (number of sunshine hours per day)	0	13·0	6·0	3·9
Mean concentration of pollutants (μm/m^3^)	1	72·0	27·4	10·9

* Median = 0 mm (interquartile range: 0–2·0 mm).

The number of ILI and SARI cases tends to be higher between July 5, 2009, and August 22, 2009. In this period, the mean temperatures (Tmin: 9·52 ± 3·89°C and Tmax: 18·9 ± 4·92°C) and the sunshine duration (4·13 ± 3·31 hours of sun per day) were lower, as expected in winter. The rainfall tends to be higher than the median calculated for the entire year (0 mm, IQR: 0–5·4 mm). In addition, the absolute humidity (AH; 7·8 ± 2·1 g/kg) and mean concentration of pollutants (20·0 ± 7·0 μm/m^3^) were lower in this period compared with the annual values.

Table 5 shows multivariate logistic regression model for IFI and PCR. We included 255 patients in logistic regression for IFI and 180 patients in logistic regression for PCR, because we have climate data only for the first year of study. IFI positivity was statistically associated with AH (OR: 0·72; 95% CI 0·59–0·86), use of air conditioning (OR: 4·16; 95% CI 1·45–11·83), and presence of mold in home (OR: 2·95; 95% CI 1·10–8·29). On the other hand, PCR positivity was statistically associated with use of air conditioning (OR: 2·27; 95% CI 1·04–4·97), average temperature (OR: 0·92; 95% CI 0·86–0·98), and mean concentration of pollutants (OR: 1·04; 95% CI 1·00–1·08).

**Table 5:** Multivariate logistic regression model for IFI and PCR (IFI: November 2008–2009; *n* = 255 and PCR: November 2008 to September 2009; *n* = 180)

Variable	OR	95% CI
IFI positive		
Absolute humidity	0·72	0·59–0·86
Air conditioning	4·16	1·45–11·8
Mold in home	2·95	1·10–8·29
PCR positive		
Air conditioning	2·27	1·04–4·97
Average temperature	0·92	0·86–0·98
Mean concentration of pollutants	1·04	1·00–1·08

OR, odds ratio; CI, confidence interval.

The multivariate time-series models for ILI and SARI cases are summarized in Table 6. Sunshine duration was the only independent covariate that was significantly associated with the frequency of ILI cases. The β-coefficient for this parameter was negative, indicating increasing ILI frequency with decreasing sunshine duration. In the model for SARI cases, the following variables proved to be significant: mean temperature (β = 0·399; *P* = 0·025), sunshine duration (β = −0·392; *P* = 0·007), RH (β = −0·098; *P* = 0·05), and mean concentration of pollutants (β = −0·079; *P* = 0·018).

**Table 6:** Multivariate time-series models for ILI and SARI (ILI: November 2008–2009; *n* = 1·321 and SARI: November 2008–2009; *n* = 845)

	β	SE	*P* value
ILI			
ARIMA (1,1,3) – ARCH (2) model			
AR1	−0·968	0·024	<0·001
MA1	0·421	0·056	<0·001
MA2	−0·638	0·046	<0·001
MA3	−0·172	0·049	<0·001
A0	12·509	0·282	<0·001
A1	0·218	0·082	0·007
A2	0·535	0·074	<0·001
Sunshine duration	−0·395	0·211	0·03
AIC		2264·2	
BIC		2287·6	
Jarque–Bera test			<0·001
Ljung–Box test			0·973
SARI			
ARIMA (0,1,3) – ARCH (4) model			
MA1	−0·703	0·053	<0·001
MA2	−0·176	0·059	0·003
MA3	0·111	0·049	0·026
A0	1·551	0·269	<0·001
A1	0·131	0·087	0·067
A2	0·395	0·103	<0·001
A3	0·241	0·078	<0·001
A4	0·158	0·078	0·021
Mean temperature	0·399	0·209	0·025
Sunshine duration	−0·392	0·160	0·007
Relative humidity	−0·098	0·059	0·05
Mean concentration of pollutants	−0·079	0·037	0·018
AIC		1857·2	
BIC		1888·3	
Jarque–Bera test			0·014
Ljung–Box test			0·949

ILI, influenza-like illness; SARI, severe acute respiratory infection; ARIMA, autoregressive integrated moving average; ARCH, autoregressive conditional heteroskedasticity; AIC, Akaike information criterion; BIC, Bayesian information criterion.

## Discussion

Acute RVIs are responsible for causing significant levels of morbidity and mortality. The most common respiratory syndrome caused by these pathogens is ILI. A more severe presentation, named SARI, was also related to some RVs.^16^,^17^ In this study, we have examined the relationship between ILI and SARI cases, meteorological variables, and air pollution using multivariate time-series analyses. We found that ILI cases were inversely correlated with sunshine duration. In addition, SARI cases were significantly associated with mean temperature, sunshine duration, RH, and concentration of pollutants.

Seasonal cycles of infectious diseases have been attributed to changes in atmospheric variables, the prevalence or virulence of the pathogen, or the behavior of the host.^18^ Earlier investigations have demonstrated that lower temperatures and sunshine duration, conditions usually encountered in winter, were associated with admissions for RVI.^12^,^19^ Temperature was found to be highly inversely correlated with RSV, influenza A, and adenovirus frequency.^12^ Interestingly, we found a positive correlation between temperature and SARI cases. One possible explanation is that it was demonstrated that for every one degree Celsius rise in temperature, the risk of premature death and acute morbidity especially among respiratory patients is up to six times higher than in the rest of the population. Second, evidence is emerging that increasing temperature is associated with increases in air pollution, especially ground-level ozone, and can amplify the adverse effects of poor air quality.^20^ Taking this evidence into account, we could expect that higher temperatures may have increased concentration of pollutants, leading to more SARI cases. However, our data showed a decrease in air pollution during the months with a higher prevalence of SARI. The third hypothesis to explain the relationship between higher temperatures and SARI cases was related to El Niño Southern Oscillation (ENSO) phenomenon. ENSO undergoes cycles between warm phases (El Niño episodes) and reverse cold phases (La Niña episodes). In the southern region of Brazil, this phenomenon is associated with elevated temperatures and rainfall, especially in spring and in the period between May and July. Previous reports have determined that El Niño events were associated with increased hospitalizations and more severe influenza epidemics.^21^,^22^

Severe acute respiratory infection cases were found to be negatively related to RH in our study. Previous studies have demonstrated that higher RH decreases the survival of lipid-enveloped virus, like influenza A, influenza B, RSV, and PIV.^23^–^25^ The use of indoor heating in winter lowers the RH; breathing dry air could cause desiccation of the nasal mucosa, epithelial damage, and reduced mucociliary clearance, increasing the host susceptibility to RVIs.^19^ However, even in tropical regions with humid climate (RH >70%), a higher activity of influenza can be found. This observation could be explained by the variation of viral stability in different RH levels. The stability of aerosolized influenza virions is maximal at lower RH (20–40%), moderate at higher RH (60–80%), and minimum at a mid-range RH (50%).^23^

In a multivariate logistic regression model for IFI-positive patients, we found that AH was a protect factor for RVI. A recent study suggested that AH may better correlate with influenza virus survival and transmission. Unlike RH, AH measures the actual water vapor content of air irrespective of temperature and has a prominent wintertime low, both indoor and outdoor. Such findings suggest that humidification measures could be helpful decreasing survival and transmissibility of influenza.^26^

Air pollution has been associated with adverse health outcomes. Studies have suggested acute effects causing respiratory symptoms, cardiovascular events, hospital admissions, and mortality. Although the available evidences indicate associations between exposure to pollutants and increased risk of RVI, potential mechanisms mediating these effects are largely unexplored.^27^,^28^ Surprisingly, our results showed that SARI cases were associated with a decrease in mean concentration of pollutants. In fact, this could be a reflection of higher rainfall in the same period, as rain acts washing out or scattering pollutants from atmosphere.^29^ On the other hand, we cannot exclude an effect of indoor pollution. In the last years, indoor pollution has been recognized as an emerging health problem, as about 90% of our time is spent indoors where we are exposed to chemical and biological contaminants.^30^ We estimated indoor pollution indirectly in our study, questioning patients about the use of wood stoves and air conditioning, and the presence of mold in home. Our findings suggested that IFI-positive patients were more prone to live in a residence with mold growth. Dampness and mold are two important sources of indoor pollution, consistently associated with respiratory symptoms. Home dampness may be a marker for mold growth, dust mites, endotoxins, and reduced ventilation, which could increase concentrations of indoor pollutants.^31^ Cough, wheezing, and upper respiratory symptoms were associated with dampness and mold in a meta-analysis.^32^ According to these results, the prevalence of cough and wheezing was higher in patients with mold in home and IFI positive.

Air conditioning was also positively related to IFI test in this study. Air conditioning use was associated with fewer hospital admissions for cardiovascular diseases, chronic obstructive pulmonary disease, and pneumonia on days with high concentrations of PM_10_,^33^ as individuals are less exposed to outdoor pollutants. Nevertheless, the majority of virus transmission occurs within indoor, air-conditioned (i.e., cooler, lower humidity) environments that favor airborne virus survival and transmission.^24^,^25^ In hot and humid conditions, indoor transmission in air conditioning environments may account for most of the transmission.^34^

We found a prevalence of 22% of RV, which is higher than that previous studies have demonstrated (between 12% and 15% in adults).^12^,^13^ Moreover, the length of stay was lower in our IFI- and/or PCR-positive patients. This finding is consistent with existing knowledge that virus identification allows the prompt initiation of therapy when indicated and avoids the unnecessary use of antibiotics, decreasing the length of hospital stay.

The present study has some limitations. First, it was based on data collected from a single center, which may have potential biases because of the characteristics of the catchment population, like vaccination coverage. Second, it is also important to note that this investigation was performed in a group of hospitalized patients, which is a bias toward the most severe disease cases. Additionally, we do not have the concentrations of individual air pollutants, but it is implausible to reliably separate the effects of air pollutants because they frequently react with each other, sometimes potentiating individual effects.^10^,^35^ The short study period should also be considered a limitation. Finally, the use of molecular techniques (PCR) in all study patients could be useful, increasing the number of viruses detected, as limited sensitivity of IFI method is well known.^10^,^36^ Despite these limitations, this is the first study, to our knowledge, to analyze the relationship between RV, meteorological parameters, and air pollution in an adult population.

In conclusion, we found that in adult patients admitted to ER with respiratory complaints, at least 22% of infections were caused by RV. The correlations found among meteorological variables, air pollution, ILI/SARI cases, and RV demonstrated the relevance of climate factors as significant underlying contributors to the prevalence of RVI in a temperate region. There is still a need of additional investigations to clarify and confirm these data, perhaps using longer time-series observations.
